# Efficacy and safety of brand-risperidone versus similar-risperidone in elderly patients with neuropsychiatric disorders: A retrospective study

**DOI:** 10.1590/S1980-57642010DN40100012

**Published:** 2010

**Authors:** Jefferson Cunha Folquitto, Sérgio Barbosa de Barros, Jony Arrais Pinto Junior, Cássio M.C. Bottino

**Affiliations:** 1 Projeto Terceira Idade (PROTER), Instituto e Departamento de Psiquiatria, Faculdade de Medicina da Universidade de São Paulo, São Paulo, Brasil.; 2 Departamento de Estatística, Universidade de São Paulo, São Paulo, Brasil, Projeto Terceira Idade (PROTER), Instituto e Departamento de Psiquiatria, Faculdade de Medicina da Universidade de São Paulo, São Paulo, Brasil.

**Keywords:** risperidone, elderly, treatment, efficacy, side effects

## Abstract

To compare the efficacy and tolerability of brand-risperidone against similar-risperidone in elderly outpatients.

**Method:**

The medical files of 16 elderly outpatients from the IPq-HCFMUSP treated with two formulations of risperidone (brand and similar) between July/1999 and February/2000 were reviewed. Two independent raters, using the Clinical Global Impression scale, evaluated the efficacy of the treatment with risperidone and the frequency of adverse effects.

**Results:**

Comparing October/1999 to November/1999, Rater 1 observed a trend (p=0.059) and Rater 2 found a statistically significant difference, in favor of the brand-risperidone group (p=0.014). Comparing October/1999 to February/2000, Rater 1 observed no statistically significant difference (p=0.190), but the Rater 2 found a statistically significant difference in favor of the brand-risperidone group (p=0.029). Comparing November/1999 to February/2000, both raters found no statistically significant differences between both risperidone formulations. Regarding adverse effects, a statistically significant difference (p=0.046) was found in favor of the patients treated with brand-risperidone.

**Conclusions:**

The risperidone-reference, compared to similar-risperidone, showed a trend toward greater efficacy and tolerability.

## Abstract (pt)

Comparar a eficácia e a tolerabilidade da risperidona-referência versus a risperidona-similar em pacientes idosos.

**Métodos:**

Os prontuários de 16 pacientes do IPq-HCFMUSP tratados com duas formulações de risperidona (referência e similar) entre julho/1999 e fevereiro/2000 foram revisados. Dois examinadores independentes, utilizando a escala de Impressão Clínica Global, avaliaram a eficácia do tratamento com risperidona e a freqüência de efeitos colaterais.

**Resultados:**

Comparando outubro/1999 e novembro/1999, o avaliador 1 observou uma tendência (p=0,059) e o examinador 2 encontrou uma diferença estatisticamente significativa a favor da risperidona-referência (p=0,014). Comparando outubro/99 com fevereiro/2000, o avaliador 1 não encontrou diferença estatisticamente significativa (p=0,190), mas o examinador 2 encontrou uma diferença estatisticamente significativa a favor da risperidona-referência (p=0,029). Entre novembro/1999 e fevereiro/2000, ambos examinadores não encontraram diferença significativas entre as formulações. Quanto aos efeitos colaterais, houve diferença significativa (p=0,046) a favor do grupo risperidona-referência.

**Conclusões:**

A risperidona-referência, quando comparada à risperidona-similar, mostrou tendência a maior eficácia e tolerabilidade.

Risperidone is a second generation antipsychotic drug, with antagonistic activity on the dopaminergic (D_1_, D_2_, D_3_, D_4_), serotoninergic (5-HT_1A_, 5-HT_2A_ and 5-HT_2C_), adrenergic α-1 and α-2, and histaminergic receptors (H_1_).^1^ Risperidone has shown efficacy in the treatment of schizophrenic patients, presenting less adverse effects compared to first generation antipsychotics.^2-9^ For the general population, the currently recommended mean dose of risperidone is 4 mg/day; larger doses can cause greater incidence of adverse effects, especially extra-pyramidal symptoms.^10^

Risperidone was studied in the management of other psychiatric disorders demonstrating efficacy in the treatment of bipolar affective disorders,^11-13^ and also in the treatment of the behavioral and psychological symptoms of patients with dementia.^14-18^ In patients with dementia, risperidone was also better tolerated than the first generation antipsychotics, especially at low doses (average of 1-2 mg/day). Therefore, the drug was considered by expert consensus to be one of the best choices for the treatment of agitation and delirium in patients with dementia.^17^

Katz et al.^14^ studied 625 patients with Alzheimer's disease (73%), vascular dementia (15%), or mixed dementia (12%), that presented psychological symptoms and behavioral alterations. Subjects were randomized to receive placebo or risperidone 0.5 mg, 1.0 mg or 2.0 mg/day for 12 weeks. Scores on the Behavioral Pathology in Alzheimer's Disease Rating Scale (BEHAVE-AD) were significativelly lower in patients taking 1-2 mg risperidone compared to the placebo group at the end of the 12-week follow-up period.^14^

In Brazil, Laks et al.^18^ showed that patients with dementia (n=26) treated with risperidone oral solution (starting dose of 0.25 mg with increments of 0.25 mg) presented a 26% reduction in agitation and no cardiovascular side effects in the dose range of 1.0 to 1.25 mg.^18^

Recent reports have associated a significant increase in the mortality rate (3.5 vs 1.5%) and in the risk of stroke (1.3 vs. 0.4%) in elderly demented patients to the use of risperidone.^19-20^

Currently, brand-risperidone and several generic and similar-risperidones are available in the Brazilian pharmaceutical market. Some studies have compared the bioequivalence of these different formulations in recent years, but there is scant data in literature comparing the clinical efficacy of these compounds.

The aim of the present retrospective study was to compare the efficacy and tolerability of brand-risperidone used at the "Instituto de Psiquiatria do Hospital das Clinicas da Faculdade de Medicina da Universidade de São Paulo" (IPq-HCFMUSP) up to October 1999, against a similar-risperidone formulation prescribed from October 1999 for older patients with psychiatric disorders. This change was compulsory due to hospital procedures and affected all patients.

## Methods

The medical files of the patients attended at the IPq-HCFMUSP outpatient units between July/1999 and February/2000 were examined. All patients who used risperidone were identified in a preliminary selection. Subsequently, the sample selected for this study had to fulfill the following inclusion criteria: presence of at least one of the following neuropsychiatric symptoms - hallucination/delirious, agitation/aggressiveness, bizarre behavior and disorganization; age 60 years or above; and outpatient treatment from July 1999 to March 2000. The neuropsychiatric diagnosis was made according to ICD-10 criteria (OMS, 1993).^20^ The exclusion criteria were hospitalization due to psychiatric aspects or due to clinical comorbidity before or after the change of medication, and the occurrence of important social and physical events (mourning, sickness, accidents) that could have had an impact on the treatment. When comparing the side effects, patients using anti-cholinergic drugs (e.g. biperidene) were excluded.

The clinical response in terms of the presence of behavioral and psychological symptoms, was verified in 3 visits (October/1999, November/1999 and February/2000). To obtain this information, 2 independent psychiatrists who were not blinded to the medications used, applied the first item of the Clinical Global Impression scale (CGI).^21^ This scale has two items, with scores ranging from 1 to 7, to rate individuals' clinical condition from healthy to severely ill and to address the rate of clinical improvement under the treatment.^21^

The statistical analysis was performed using the statistical package SPSS14.0 for Windows. Initially, the socio-demographic characteristics of the sample were presented. The inter-rater reliability was measured using the weighted kappa index for each visit. The pharmacological response was evaluated in the months immediately before and after the period when the brand-risperidone was changed to similar-risperidone (October/1999 versus November/1999, and October/1999 versus February/2000); and during another period (November/1999 versus February/2000) after the change over. These comparisons were made using the Wilcoxon nonparametric test. The frequency of adverse effects before and after the change of risperidone formulations were also analyzed using the Wilcoxon test, and the mean dose of risperidone before and after the medication change was compared using student’s t test.

The present study was approved by the research ethics committee of the “Hospital das Clínicas da Faculdade de Medicina da Universidade de São Paulo” (nr. 0665/07).

## Results

Eighty-nine patients received risperidone between July/99 and March/2000. Of these patients, only 16 were eligible according to the inclusion criteria (age=73.5±6.5 years; female=50%). Of the selected patients, 13 had a diagnosis of dementia (81.3%), 2 of depression (12.5%) and 1 of schizophrenia (6.2%), according to ICD-10 criteria.^22^ The socio-demographic characteristics of the sample are shown in Table 1.

**Table 1:** Characteristics of the sample.

Patient	Age**	Gender***	Rater 1*				Rater 2*				Adverse effects^#^			Medication^##^	
			**Oct 1999**	**Nov 1999**	**Feb 2000**		**Oct 1999**	**Nov 1999**	**Feb 2000**		**Oct 1999 visits**	**Nov 1999 and** **Feb 2000 visits**		**Oct 1999 visits**	**Nov 1999 and Feb 2000 visits**
1	80	M	4	6	4		5	6	4		1	2		3.5	3.5
2	72	F	5	5	6		5	5	6		0	0		1.0	1.0
3	74	M	5	5	6		5	5	6		0	1		4.0	4.0
4	76	F	5	5	6		5	5	5		0	0		3.0	3.0
5	71	F	4	4	3		3	4	4		0	0		4.0	4.0
6	63	M	6	6	6		6	6	6		0	0		1.0	2.0
7	71	M	4	4	4		4	4	4		NA	NA		1.0	1.0
8	88	M	4	4	6		4	4	6		0	0		1.5	1.5
9	80	F	6	6	5		6	6	5		0	0		2.0	3.0
10	61	F	5	6	5		5	6	6		0	1		3.5	3.5
11	77	F	5	6	6		5	6	6		0	0		1.0	1.0
12	77	F	4	5	6		5	6	6		1	1		1.0	1.0
13	71	M	4	4	3		4	4	4		1	2		1.0	1.0
14	67	F	5	5	5		5	5	5		Bip	Bip		2.0	2.0
15	76	M	5	5	5		5	5	6		1	1		4.0	3.0
16	72	M	5	5	5		4	5	6		1	1		3.0	4.0

* Score for the first item of the CGI;

** In years;

*** M, male; F, female;

# NA, not available, Bip, use of biperidene; 0, absence of side effects; 1, mild side effects; 2, moderate to severe side effects;

## mean dose: Before, dose used in visits in October/99; After, mean dose used in visits in November/1999 and February/2000.

The inter-rater reliability, evaluated using the weighted kappa index, ranged from 0.628 to 0.925, with the lower value corresponding to the February/2000 visit and the highest value in the November/1999 visit.

**Figure 1:**
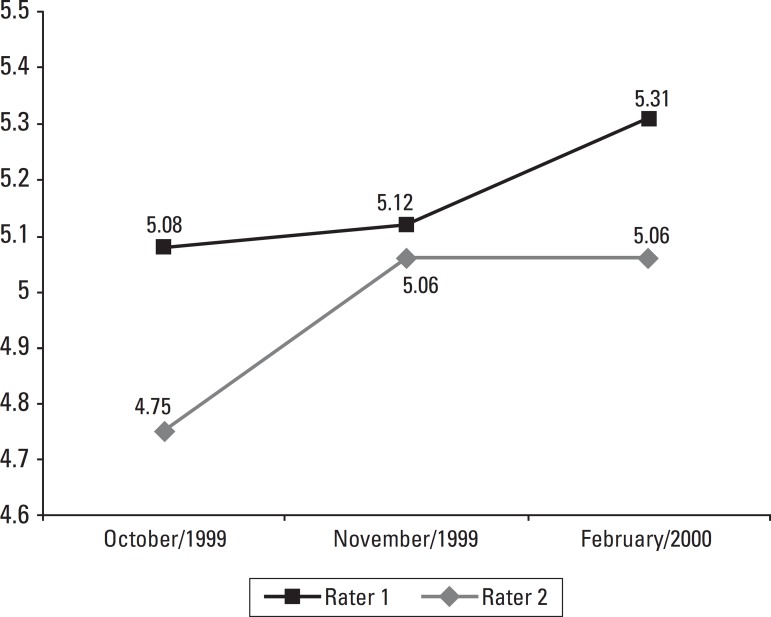
Mean CGI in October/1999, November/1999 and February/2000.

Regarding the efficacy of the risperidone formulations, as evaluated by the CGI, Rater 1 showed a tendency to report better response in the month before the change (Z=–1.89, p=0.059). However, Rater 2 observed a significant difference in favor of the brand-risperidone (Z= –2.44, p=0.014). Comparing October to February, the results of Rater 1 showed no statistical difference (Z= –1.31; p>0.05), but a significant difference was found by Rater 2 (Z= –2.17, p=0.029). In the comparison between November/99 and February/2000, both raters found no statistically significant differences (Rater1: Z=0.00, p>0.05; Rater2: Z= –0.79, p>0.05). The mean CGI values reported by each rater are depicted in Figure 1.

When patients without dementia were removed from the sample, only a single statistical change was observed, in the comparison between October/1999 and February/2000, in which the Rater 2 found only a trend toward statistical difference in favour of the brand-risperidone group.

For the adverse effects analysis, only 14 patients were considered since 2 subjects were excluded: one due to the absence of data in their medical records, and the other due to use of anticolinergic medication (Biperidene) to alleviate adverse effects. Of the 14 patients analyzed, 4 presented adverse effects before the change of risperidone formulations (2 patients with symptoms of induced parkinsonism, and 2 with tardive dyskinesia). After the medication change, 2 of these 4 patients showed a worsening in their condition: 1 presented tardive dyskinesia, 1 induced parkinsonism and 2 remained stable. In the group of 10 patients that did not present adverse effects initially, 2 started to show induced parkinsonism, and 1 presented dyskinetic movements and sedation. On the Wilcoxon test, a significantly higher frequency of adverse effects associated with similar-risperidone was observed (Z= –2.00, p=0.046).

With regard to the mean dose of risperidone, there was no statistically significant difference before and after the change from brand-risperidone to similar-risperidone (t= –0.28, p=0.77).

## Discussion

In the Brazilian pharmaceutical market, 3 kinds of drug formulas are available: the brand formula, which is the medication originally developed by the pharmaceutical industry, having passed various tests before being introduced onto the market, and possessing well-known pharmacological characteristics; the generic formula produced by laboratories required to perform tests of bioequivalence versus the reference drugs; and the similar formula, drugs for which bioequivalence testing was not required by the Federal Government Agency before receiving approval to be sold in the market.^23,24^

Since it is not mandatory to perform bioequivalence testing to approve similar formulations, there is no independent data to compare with the brand formulations. Consequently, the efficacy of similar category drugs may hypothetically differ. To our knowledge, there are few published studies in Brazil regarding the bioequivalence of brand and similar psychotropic formulations, and such trials involving risperidone. In the international literature, Borgherini (2003) found few studies comparing brand-name and generic psychotropic drugs, reporting that many of these studies showed significant discrepancies between the different compounds. Some studies comparing brand-name and generic Clozapine found a better clinical response for the brand-name Clozapine.^25^ Van Os et al.^26^ studying 32 healthy volunteers found a lack of bioequivalence between a generic oral solution of risperidone and brand-risperidone tablets.^26^ In Chile, Gaete et al.^25^ compared the bioavailability of the risperidone-similar to the brand-risperidone in 12 healthy volunteers. Applying the methods suggested by the Food and Drug Administration (FDA) of the United States (90% confidence interval for the difference of log transformed mean pharmacokinetic parameters), the authors found these two presentations of risperidone to lack bioequivalence.^27^ Crawford et al. (1996) observed negative health benefit in approximately 10% of a sample of epilepsy patients after change of reference-medication to generic-medication, besides increased social costs.^28^ However, some clinical studies in patients with epilepsy found no difference in efficacy between reference and generic drugs.^29-30^ Vadney and Kraushaar (1997) observed no statistically significant changes in seizures and blood levels in individuals with mental retardation after switching from reference to generic Valproic Acid. ^29^

In the present study, a statistical trend toward lower efficacy of the similar-risperidone in comparison to the brand-risperidone was observed. A higher frequency of adverse effects associated with the change from brand-risperidone to similar-risperidone was also observed.

It is noteworthy that our study presented several methodological limitations, such as the small sample size (n=16), which substantially reduces its statistical power. To date, studies available in the literature comparing similar and reference formulations of psychotropic drugs have involved only small patient samples. Although our sample was predominantly composed of Alzheimer’s disease patients (81.3%), it also comprised patients with schizophrenia and depressive disorder. When only patients with dementia were analyzed, the results did not change substantially. The retrospective design of the study (which could have biased the CGI scoring), the use of non-blinded raters, and the lack of a control group constitute other potential limitations. We also question whether the greater presence of side effects associated with similar-risperidone was not due to increased physical activity in the patients compared to the reference-risperidone users. Finally, the poorer response found after the change over from brand-risperidone to similar-risperidone, could not be disentangled from the natural tendency to deteriorate, since most of the patients had a diagnosis of dementia, typically a progressive disease.

To conclude, our study found a trend toward higher efficacy and less extrapiramidal side effects associated to the use of brand-risperidone compared to similar-risperidone in elderly outpatients with psychiatric disorders. These preliminary findings call into question the Brazilian laws for medication control that allow the approval of similar drugs without rigorous testing of their pharmacological properties. In any event, it is necessary to perform additional studies which employ rigorous methodologies (prospective, blinded, with control group) to further investigate this question, addressing not only bioequivalence but also clinical equivalence, in terms of efficacy and adverse effects, of psychotropic formulas.
